# P-1686. Deep Learning Prediction Model for Staphylococcus aureus Positivity and Antibiotic Susceptibility Patterns Using A Large Longitudinal Electronic Health Record Dataset

**DOI:** 10.1093/ofid/ofaf695.1860

**Published:** 2026-01-11

**Authors:** Francis Ifiora, Laila Bekhet, Ziqian Xie, Marilyn Niravath, Stephen Jones, Cesar A Arias, Degui Zhi, Masayuki Nigo

**Affiliations:** Houston Methodist Research Institute, Houston, Texas; School of Biomedical Informatics, University of Texas Health Science Center at Houston, Houston, Texas; UTHealth School of Biomedical Informatics, Houston, Texas; Houston Methodist, Houston, Texas; Houston Methodist Hospital, Houston, Texas; Houston Methodist and Weill Cornell Medical College, Houston, TX; UTHealth School of Biomedical Informatics, Houston, Texas; Houston Methodist Hospital, Houston, Texas

## Abstract

**Background:**

*Staphylococcus aureus* causes a range of infections, including bacteremia, pneumonia, and bone/joint infections, and exhibits variable antimicrobial resistance. Predicting both the infection source and resistance patterns is critical for clinical decision-making. However, existing models often focus solely on detecting resistant strains like MRSA, without addressing the infection source or broader resistance profiles. We developed a deep learning model to predict both the source and resistance patterns of *S. aureus*.

**Table 1:**
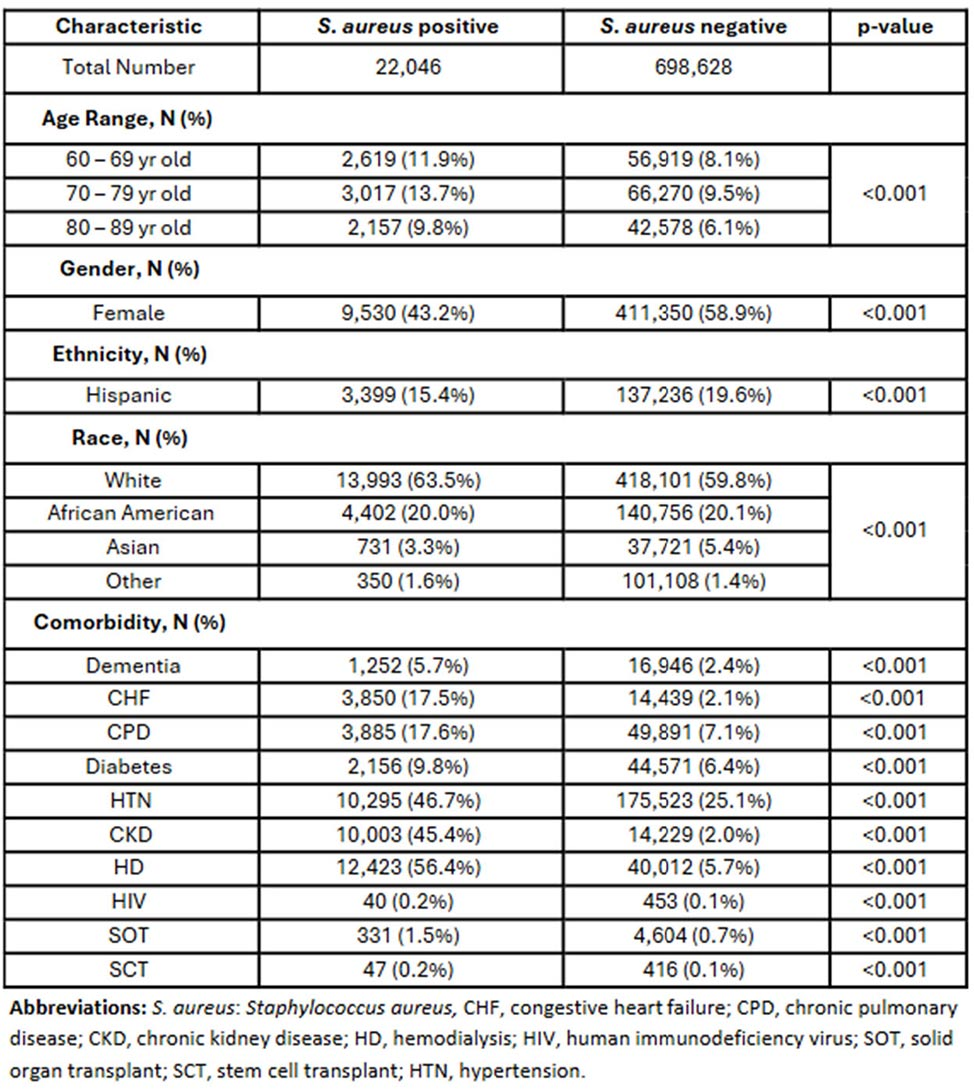
Patient characteristics of the study population stratified by Staphylococcus aureus status.

**Table 2:**
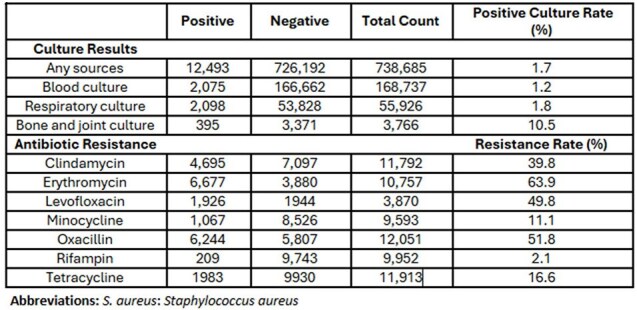
Summary of culture positivity and antibiotic resistance rates in cohort.

**Methods:**

We retrospectively collected time-sequenced electronic health record (EHR) data of patients who had microbiology cultures at Houston Methodist Hospital System (eight hospitals) from May 2016 to June 2023. Data were split into training, validation, and test sets (70:10:20). The model was developed in two steps: (1) predicting the presence and source of *S. aureus*, and (2) for positive cases, predicting antimicrobial susceptibility. A 7-day prediction window was used to align with clinical decision-making; culture events >7 days apart were treated as separate predictions.

**Table 3.**
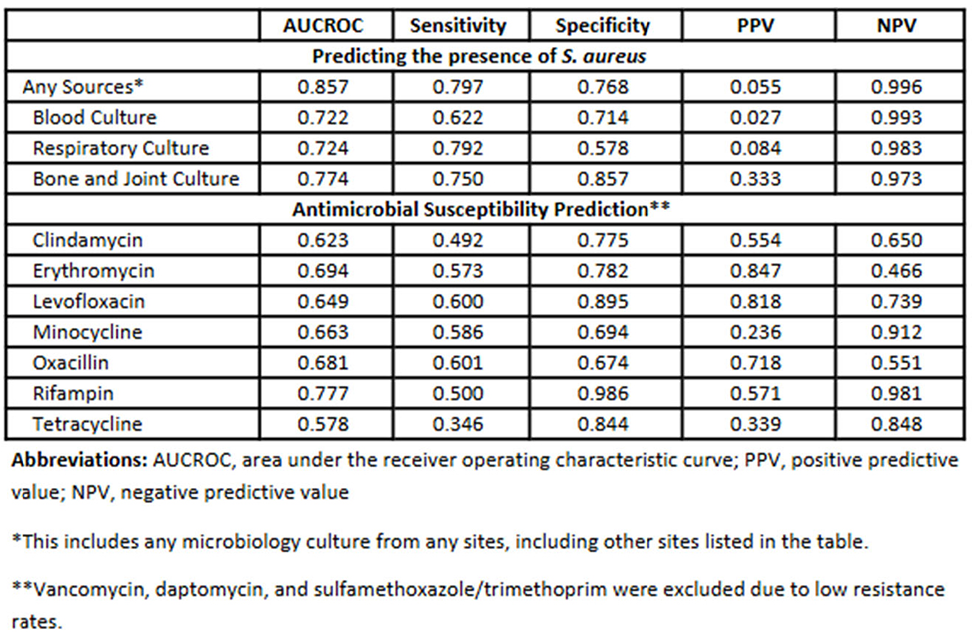
Model Performance

**Results:**

We analyzed 956,428 culture events from 720,674 patients, of which 22,046 were culture-positive for *S. aureus*. Table 1 presents patient characteristics stratified by the positivity of *S. aureus*. Compared to negative cases, *S. aureus*-positive patients were more likely to be male, aged 70–79, and have comorbidities. Table 2 summarizes positivity rates from clinical cultures and resistance patterns, with high resistance rates observed for Erythromycin (63.9%), Oxacillin (51.8%), and Clindamycin (39.8%). Table 3 reports model performance. The AUROC for predicting *S. aureus* presence from any culture source was 0.857, with an NPV of 0.996. Antibiotic resistance models achieved AUROCs up to 0.777 (rifampin), followed by oxacillin (AUROC of 0.681).

**Conclusion:**

Our deep learning model demonstrated high performance in identifying *S. aureus* from any microbiology culture and achieved moderate to high accuracy in predicting the selected sources. The model also showed moderate accuracy in predicting antimicrobial resistance patterns. While these results highlight the model’s potential, further refinement is needed to enhance its performance.

**Disclosures:**

All Authors: No reported disclosures

